# P300, Gray Matter Volume and Individual Characteristics Correlates in Healthy Elderly

**DOI:** 10.3389/fnagi.2019.00104

**Published:** 2019-05-03

**Authors:** Valentina Pergher, Jos Tournoy, Birgitte Schoenmakers, Marc M. Van Hulle

**Affiliations:** ^1^Laboratory for Neuro- and Psychophysiology, Department of Neurosciences, KU Leuven – University of Leuven, Leuven, Belgium; ^2^Department of Chronic Disease, Metabolism and Ageing, KU Leuven – University of Leuven, Leuven, Belgium; ^3^Academic Centre of General Practice, KU Leuven – University of Leuven, Leuven, Belgium

**Keywords:** magnetic resonance imaging, N-Back task, sex, education, P300-ERP, elderly

## Abstract

We investigated whether P300-ERP and cognitive test performance differ for age, sex, and education in two groups of healthy elderly, and verified whether any correlations exist between P300 amplitude and latency and gray matter volume using whole brain voxel-by-voxel-based mapping, controlling for age, education, sex and Total Intracranial Volume (TIV). We used 32 channel electroencephalograms (EEG) to record the P300 responses and 3T Magnetic Resonance Imaging (MRI) to determine gray matter volume. We recruited 36 native-Dutch speaking healthy older subjects, equally divided in two sub-groups of 52–64 and 65–76 years old, administered a battery of cognitive tests and recorded their demographics, EEGs and task performance; additionally, 16 adults from the second sub-group underwent an MRI scan. We found significant differences between age groups in their cognitive tests performance, P300 amplitudes for the frontal and parietal electrodes for the most difficult task, and P300 latencies for frontal, central and parietal electrodes for all three tasks difficulty levels. Interesting, sex and education affected cognitive and P300 results. Higher education was related to higher accuracy, and P300 amplitudes and shorter latencies. Moreover, females exhibited higher P300 amplitudes and shorter latencies, and better cognitive tasks performance compared to males. Additionally, for the 16 adults underwent to MRI scan, we found positive correlations between P300 characteristics in frontal, central and parietal areas and gray matter volume, controlling for demographic variables and TIV, but also showing that age, sex, and education correlate with gray matter volume. These findings provide support that age, sex, and education affect an individual’s cognitive, neurophysiological and structural characteristics, and therefore motivate the need to further investigate these in relation to P300 responses and gray matter volume in healthy elderly.

## Introduction

The increased life expectancy has led to an increase in dementia cases among older adults as age is one of the risk factors. Despite the absence of cognitive impairment, about 20–40% of 60–90 years old healthy individuals exhibit high levels of Aβ deposition in the brain and will develop dementia in the coming years (Bateman et al., 2012; Villemagne et al., 2013), exhibiting impairments in several cognitive functions, such as processing, memory, attentional control, motor, and sensory abilities (Jackson and Owsley, 2003; Tales et al., 2004).

Cognitive tests have been used to chart normal changes in cognitive performance over adult lifespan (Christensen, 2001) such as attention, performance speed, recall memory, working memory (WM), verbal fluency, reasoning, and spatial abilities (Chao and Knight, 1997; MacPherson et al., 2002; Drag and Bieliauskas, 2010). Besides age, also educational level and sex have shown to affect cognitive tests performance. Wiederholt et al. (1993) observed that male performance declines more rapidly with age and that men and women with a higher level of education perform better than those with a lower educational level. Ruff and Parker (1993) showed that sex and level of education affect performance speed. When testing healthy older adults, they observed that women were faster than men and higher educated individuals performed faster than lower educated ones in Finger Tapping and Grooved Pegboard Tests, both of which require cognitive and motor abilities. In addition to cognitive testing, an increasing number of studies showed that also EEG is a powerful tool to study the effect of aging, by providing temporal information of brain activity and related cognitive functioning. Event-related Potentials (ERPs) are characteristic sequences of positive and negative amplitude deflections that are time locked to the onset of a particular stimulus such the P300 component, a positive amplitude deflection between 250 and 500 ms, with a peak around 300 ms, elicited in response to an infrequent stimulus (“oddball”) to which the viewer pays attention. The P300 is considered to reflect several cognitive functions involved during attentional and memory tasks (Picton, 1992; Polich, 2007). It has been shown that older subjects exhibit a smaller P300 amplitude and a larger P300 latency over the midline central and parietal locations (Polich, 2007; Ashford et al., 2011), even when considered healthy, indicating that normal cognitive decline across time affects P300 responses (Polich, 2007). Furthermore, Fabiani et al. (1998) showed maximal significance for frontal electrodes for older adults, which they attributed to memory decay and reduced WM capacity. Additionally, Comerchero and Polich (1999) and Hagen et al. (2006) observed that, while for the easier cognitive task the P300 amplitude of healthy adults was larger over the parietal electrodes, the more difficult task produced a larger P300 amplitude over the frontal/central electrodes. Similar results were reported by Pietto et al. (2016) and Gironell et al. (2005) as they observed a smaller P300 amplitude and a larger P300 latency in individuals that were in 1 year diagnosed with AD, compared to healthy controls. Age is not the only factor that affects P300 magnitude and as well as that of other ERPs components. It has been shown that females are characterized by a greater P300 response than males for a relevant stimulus during an object recognition task, indicating that females might process visual information differently from males, perhaps by increased allocation of attentional resources to distracting stimuli (Steffensen et al., 2008). Furthermore, the study of Angel et al. (2010) showed that ERPs components can be affected by educational level. They found that the effect of age on ERPs responses was smaller for participants that were higher educated compared to those with a lower level of education, during the performance of a word-stem cued-recall task. These findings recall the concept of cognitive reserve and the protective role it plays during aging (Christensen et al., 2008). Kramer et al. (2004) provided support for the cognitive reserve hypothesis by showing a greater synaptic density and more complex brain networks in higher educated individuals. Alternatively, Park and Reuter-Lorenz (2009) hypothesized that older adults with a higher cognitive reserve can compensate for neurocognitive deficits by recruiting alternative brain networks and in this way enable them to perform a task with similar accuracy.

Besides P300 changes, brain volume decreases with age, as shown in several MRI studies, although not homogeneously across the brain. Changes in gray matter volume seem to be prominent in the frontal lobes (Coffey, 1994; Raz et al., 1997; Tisserand et al., 2002), providing support to the frontal theory of cognitive aging (Phillips and Della Sala, 1998) that relates changes in frontal structures and functions to cognitive deficiencies, such as attentional and memory difficulties, and lack of cognitive flexibility and control. In contrast, only a few studies on aging focus on brain volume alterations in other substructures, such as temporal and parietal structures (Grieve et al., 2005; Hutton et al., 2009; Giorgio et al., 2010; Terribilli et al., 2011). Additionally, several studies revealed that aging is not the only factor in affecting gray matter volume. Raz et al. (1997) and Good et al. (2001) showed that gray matter volume variation might be also sex-related, with a steeper trend in men. Also Witte et al. (2010) found similar differences in gray matter volume between male and female in a sample of young healthy adults. Another factor that might affect gray matter volume is educational level. Arenaza-Urquijo et al. (2013) showed that more educated individuals have greater gray matter volume in the superior temporal gyrus, insula, and anterior cingulate cortex. Also Steffener et al. (2014), using voxel-by-voxel-based morphometry (VBM) applied to the whole brain, found that changes in gray matter volume are related to both age and educational level. In particular, they showed a stronger positive relationship between larger gray matter volume and better fluid intelligence performance in more educated older adults.

Although age-related changes in P300 and gray matter volume have been studied, the relation with the intracerebral origin of the P300 component is still poorly understood. No studies have evaluated neuroanatomical correlates of P300 in healthy older adults, despite it has been shown that P300 has multiple brain generators in temporal, frontal and parietal lobes (McCarley et al., 2002). Several studies have been conducted to assess the correlations between P300 abnormalities and gray matter volume in subjects at high risk for psychosis, schizophrenic patients and individuals with post-traumatic stress disorder (Havermans et al., 1999; Araki et al., 2005; Fusar-Poli et al., 2011), but only one study examined the relationship between auditory P300 and gray matter volume in healthy adults. Ford et al. (1994), using a regional measure of gray matter volume, showed significant correlations between parietally recorded P300 during an automatically elicited attention task, where participants were presented a series of auditory stimuli with fixed inter-stimulus interval, and frontal lobe gray matter volume, and between P300 recorded during both automatic and effortful attention (i.e., with higher frequencies for target stimuli) tasks, and both frontal and parietal lobes gray matter volume. Additionally, recently it has been shown that a voxel-by-voxel whole brain analysis to assess atrophy level is more sensitive compared to a ROI analysis as the latter may not be sensitive enough to detect small changes over time (Chetelat and Baron, 2003). Additionally, the ROI method implies an *a priori* hypothesis regarding the structure to assess, which could detect only partially gray matter volume changes (Chetelat and Baron, 2003). Voxel-by-voxel-based morphometry (VBM) is an automated approach, not biased to a particular structure, that provides a comprehensive assessment of gray matter volume across the whole brain from high-resolution MRI scans (Ashburner and Friston, 2000) and has been applied to both healthy older adults (Raz et al., 1997; Good et al., 2001) and to MCI and AD patients (Frisoni et al., 2008; McDonald et al., 2009).

The study we report on is the first to evaluate visual P300-ERP component, recorded during a WM task, and gray matter volume correlates, measured using the VBM technique, in healthy aging individuals. We first assessed cognitive tests performance and P300 amplitude and latency for two age-groups, young-old and old, by considering possible sex and education effects, and then examined the relationship between P300 characteristics and gray matter volume, controlling for age, education, sex, and TIV. Based on previous evidence, we expected to see differences in cognitive, neurophysiological and structural characteristics between age groups, sex and educational level. Furthermore, we hypothesized significant correlations between P300 amplitude and latency, and gray matter volume in frontal and parietal lobes.

## Materials and Methods

We selected 36 Dutch-native speaking healthy older adults, recruited by flyers, social network, advertisements in a local newspaper and a general physician’s practice. We included in our study participants between 52 and 76 years old, equally divided in two sub-groups, ranged 52–64 years old, and 65–76 years old, with a Mini Mental State Examination (MMSE) score above 27, no history of neuropsychological or psychiatric disorders, no history of traumatic brain injury, no post-traumatic cognitive dysfunction, and with good or corrected vision. For all participants the procedure consisted of simultaneous EEG recording during a WM task performance, called N-Back task (Jaeggi et al., 2010), and for 16 subjects between 65 and 76 years old, also an anatomical MR scan. The study was conducted in KU Leuven University (Belgium), the MR imaging in the Radiology Department of its University Hospital Gasthuisberg and the EEG recordings in the Laboratory of Neuro- and Psychophysiology of the Medical School. Written informed consent was obtained from all participants in accordance with the Declaration of Helsinki. The study was approved by the Ethical Committee of Gasthuisberg Hospital.

### MR Imaging and Analysis

MR imaging was performed using a 3.0 T scanner (Philips, The Netherlands). All subjects were examined according to a standard dementia MRI protocol: axial T2-weighted images, 3D fluid-attenuated inversion recovery (FLAIR), coronal T2-weighted images with perpendicular hippocampus orientation, axial diffusion weighted imaging, T2-weighted images, and gradient-echo T1-weighted 3D images. The imaging parameters of the 3D gradient-echo T1 weighted images were: TR/TE 2300/3 ms, flip angle 9°, field of view 256 × 240, slice thickness 1 mm and 160 slices.

**FIGURE 1:**
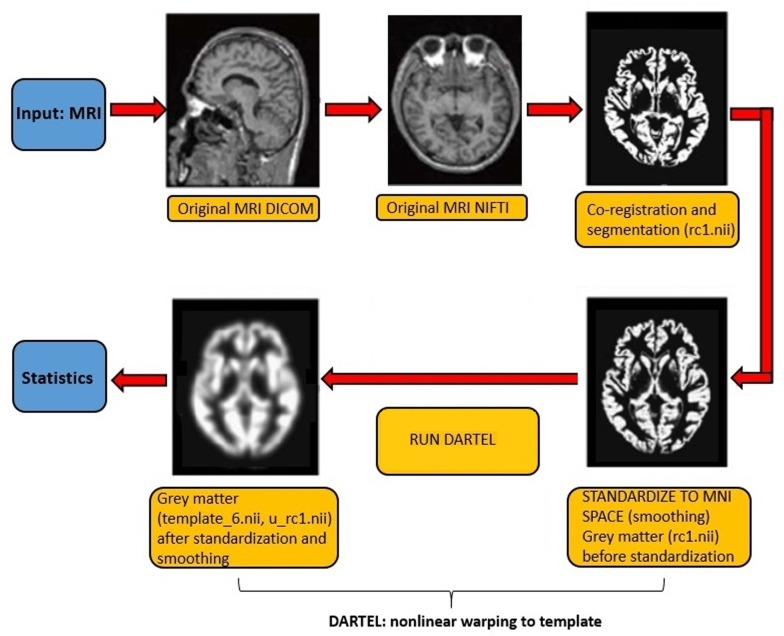
N-Back task design.

Voxel-based morphometry (VBM) maps gray matter volume on a voxel-by-voxel basis after anatomical standardization analogous to functional neuroimaging. In order to examine possible correlates of gray matter volume, we investigated both voxel activation, further called peak level, and the spatial location of the peak (further called cluster). MRI scans were analyzed with SPM12 (Statistical Parametric Mapping) ^1^ and Matlab version R2016a^2^. Processing included MRI scan segmentation (T1 images) to identify different tissue types for each subject, the creation of a template of the whole brain for all subjects using Dartel toolbox (Ashburner, 2007), the modeling of the shape of each brain using three parameters for each voxel (to increase the accuracy of subject gray matter alignment), normalization to Montreal Neurological Institute (MNI) Space of the original images to the template, the generation of smoothed (with a 5 mm isotropic Gaussian kernel), spatially normalized, and Jacobian-scaled gray matter images in MNI space (Mechelli et al., 2005; Ashburner, 2009; Kurth et al., 2015) and, finally, a linear regression analysis between gray matter correlates and frontal, central and parietal P300 component, controlling for age, education, sex and TIV, to make inferences about any differences in the data (Figure 1). Kernel smoothing was applied to estimate the age-volume relation, thus a non-parametric model as the underlying analytics is unknown and observations are noisy (Friedman et al., 2001). Furthermore, to estimate the TIV, we used an algorithm recently introduced by Malone et al. (2015), using SPM12, that integrates the probabilistic tissue class images of gray matter, white matter and cerebral spinal fluid.

The correlations between P300 characteristics and voxel-by-voxel whole brain gray matter volume were determined using a linear regression analysis. Age, education, sex, TIV and the grand-averages of P300 amplitudes and latencies were used as covariates. We used the average of the three difficulty levels as we are aiming for an initial general exploration of the possible correlations between this specific WM task (N-Back) and gray matter volume. Statistical significance was set at Family-Wise Error (FWE) uncorrected *p* < 0.001 for multiple comparison (Roiser et al., 2016) at cluster and peak levels to avoid Type I error. Anatomical regions included for analysis were defined using the Automated Anatomical Labeling (AAL) toolbox^3^ (Tzourio-Mazoyer et al., 2002). The peak coordinates were presented in MNI standard space and the results visualized using SPM12.

### EEG Acquisition and Analysis

EEG was recorded using a SynAmpsRT device (Compumedics, Australia) operating at 2 kHz sampling rate using 32 active Ag/AgCl electrodes: O1, Oz, O2, PO4, PO3, P8, P4, Pz, P3, P7, TP9, CP5, CP1, CP2, CP6, TP10, T7, C3, Cz, C4, T8, FC6, FC2, FC1, FC5, F3, Fz, F4, AF3, AF4, Fp1, Fp2. Before the N-Back task, an EOG calibration was performed using four additional electrodes to capture the effect of eye movements and blinks, following the instructions given in Croft and Barry (2000). The recorded EEG signal was re-referenced offline to the average of the two mastoid signals (average mastoid reference, TP9 and TP10), corrected for electro-oculogram (eye movement and blinking artifacts), using Croft and Barry’s aligned-artifact average (AAA) procedure (Croft and Barry, 2000), band-pass filtered in the 0.1–30 Hz range, and cut into epochs starting from 100 ms pre- till 1500 ms post-stimulus onset. Baseline correction was performed by subtracting the average of the 100 ms pre-stimulus onset activity from the 1500 ms post-stimulus onset activity. Finally, the epochs were downsampled to 100 Hz and stored for ERP component detection. Three two-way ANOVA (age-group × target, sex × target, and educational level × target) and two three-way ANOVA (age-group × sex × target, and age/group × educational level × target) were performed considering P300 amplitudes, calculated as the average over the 250–600 ms time window as it contains the largest positive-going peak of the P300 waveform (Polich, 2007), for channels Fz, Cz, and Pz, and P300 latencies, calculated as the average from stimulus onset to the point of maximum positive P300 amplitude in the same time window. P300 scalp distribution is defined as the amplitude change over the midline electrodes (Fz, Cz, Pz), which increases in magnitude from frontal to parietal electrodes (Johnson, 1993). Bonferroni correction (*p* < 0.05) was used for multiple comparison. Recorded epochs with incorrect behavioral responses (N-Back button presses, see further) were excluded from further analysis. In addition, epochs with EEG amplitudes greater than 50 μV were also excluded, as they could be motion artifacts.

### N-Back Task

**FIGURE 2:**
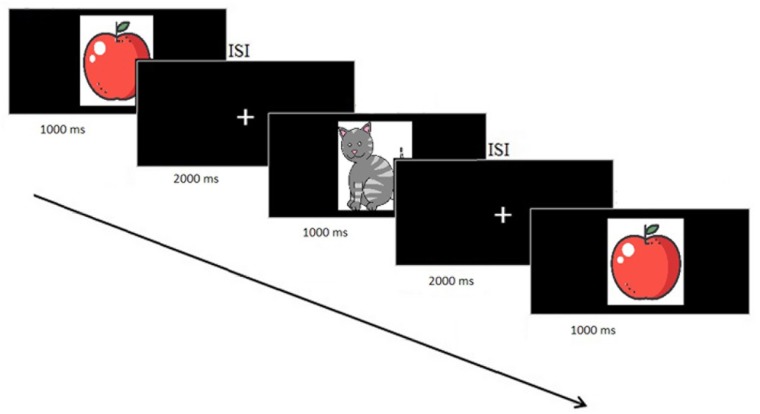
Flow diagram of the VBM procedure. The first two pictures were adapted from the study of Matsuda (2013).

Subjects performed a single session of a WM task in which a sequence of stimuli was shown and the task was to decide whether the current stimulus matched the one presented N stimuli before (N-Back task). Stimuli were presented for 1000 ms followed by a 2000 ms Inter-stimulus interval (ISI), with a jitter of ± 100 ms, during which the picture was replaced by a fixation cross (Figure 2). This was the moment where participants should press a button on the keyboard to indicate whether this stimulus matched the one shown N stimuli before. We had 33% of the pictures as targets. We started with *N* = 1 and, when the responses were for more than 70% correct, participants went to the next task difficulty level and so on until *N* = 3.

In the 1-Back the participant needed to maintain in memory only one item, and this task requests a constant updating of the new stimulus that replaces the old one. In the 2-Back task the participant needs to maintain two stimuli in memory and remember their order, and the task requests a greater replacement operation. In the 3-Back the participants needed to maintain three items in memory and uses a 3 step replacement operation. By increasing N (1-Back, 2-Back, 3-Back), the participant needed to put in increasingly more mental effort to perform the task (Gevins and Smith, 2003). By varying difficulty level, the task imposed a variable workload reflected by a change in the effort the participant needed to put into it.

Sequences with identical difficulty levels (1-back, 2-back, 3-back) were grouped into 2 min. blocks across four sessions. Each session included 2 repetitions of 3 sequences, and were presented with increasing task difficulty level (i.e., from 1- to 3-back) if the subject responded correctly for more than 70% of the cases, otherwise the task remained at the same difficulty level. In total, there were 8 blocks. For each sequence, 60 stimuli were presented in pseudorandom order.

## Results

### Participants’ Characteristics

Demographical characteristics of 36 participants are listed in Table 1. Our sample was divided in two equal-sized age sub-groups: young-old adults (*n* = 18), between 52 and 64 years, and old adults *(n* = 18), between 65 and 76 years. Furthermore, given our subjects’ years of education, we differentiated two sub-groups based on educational level: low (≤9 years, *N* = 14) and high (>9 years, *N* = 22). Before N-Back task performance, we collected additional demographic information such as sex and educational level, and administered cognitive tests to measure and compare the cognitive functioning between the sub-groups. The battery of cognitive tests we used included: MMSE (Folstein et al., 1975), Digit Span (Wechsler, 1945), Stroop (Stroop, 1935), COWAT (Ivnik et al., 1996), VAT (Lindeboom et al., 2002), TMT A and B (Reitan, 1958), Raven (Raven, 1936), TOVA (Greenberg and Waldmant, 1993), and CORSI (Corsi, 1972) tests. We used *t*-test analysis to investigate differences between sub-groups for age, sex and educational level.

**Table 1:** Characteristics of healthy older subjects.

	Age				Sex				Education			
	Young-old (*N* = 18)		Old (*N* = 18)		Female (*N* = 16)		Male (*N* = 20)		High (*N* = 22)		Low (*N* = 14)	
Variables	Mean	*SD*	Mean	*SD*	Mean	*SD*	Mean	*SD*	Mean	*SD*	Mean	*SD*
Age	58.92	4.63	67.79	3.19	63.1	3.92	62.84	6.51	62.17	5.9	64.67	4.46
Education	8.67	2.66	9	4.15	8.67	2.13	9.84	3.25	11.25	2.67	6.75	4.49
Sex	(9 M)	–	(12 M)	–	–	–	–	–	(12 M)	–	(7 M)	–
MMSE	29.72^∗^	0.46	29^∗^	1.37	29.66	0.49	29.53	0.64	29.45	1.21	29.64	0.5
Digit Span Forward	7.78^∗^	2.15	5.89^∗^	2.35	7.4	2.44	6.8	2.21	7.82^∗^	2.23	5.45^∗^	1.86
Digit Span Backward	6.72	2.4	6.05	1.92	7.33^∗^	1.91	5.73^∗^	2.28	7.54 ^∗∗^	2.38	5.54^∗∗^	1.03
COWAT	14.53	2.95	13.27	4.1	15.26^∗^	4.97	15.59^∗^	9.94	15	4.9	12.96	2.58
VAT	4.94	1.43	4.83	1.38	5.13	1.24	4.87	1.36	5.18	1.25	4.54	1.63
Stroop	99.78	0.55	99.7	0.47	99.8	0.41	99.73	0.59	99.82	0.4	99.64	0.67
TMT-A and B	1.89	0.32	1.88	0.32	1.73	0.45	1.75	0.39	1.87	0.38	1.81	0.47
CORSI	8^∗∗^	1.19	6.36^∗∗^	1.94	6.8	1.82	7.9	1.73	7.45	1.75	6.91	2.26
Raven	46.44	12.01	41.89	10.37	44.66	11.14	45	10.97	52.45	5.37	40.18	10.79
TOVA	111.88	3.97	112.72	5.31	114.07	3.99	110.33	4.92	112.91	3.33	111.09	4.44
N-Back	2.03^∗∗^	2.09	0.88^∗∗^	1.35	1.52	1.86	1.57	1.88	2.09	1.75	0.96	1.59

*MMSE, Mini Mental State Examination; COWAT, Controlled Oral Association Test; VAT, Visual Association Test; TMT, Trial Making Test. Significant differences for ^∗^p < 0.05, ^∗∗^p < 0.01.*

### Behavioral Results

#### Cognitive Evaluation

**FIGURE 3:**
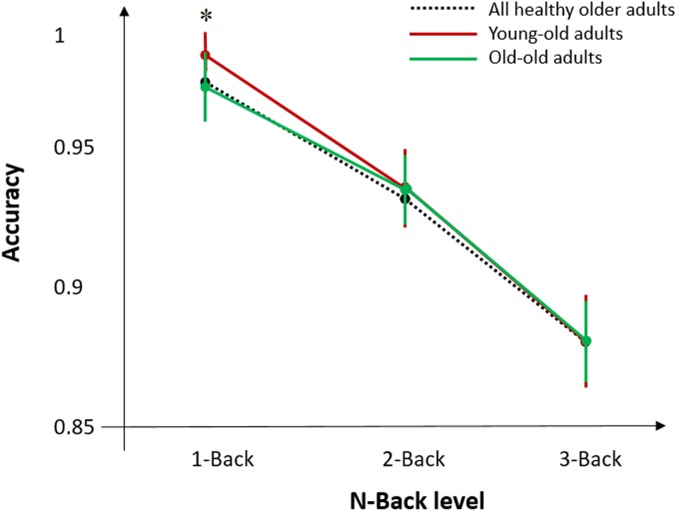
Mean accuracy during N-Back task for all healthy older adults and the two age-groups of young-old and old adults. Error bars denote standard error of the mean across subjects. Asterisks (^∗^) indicate significant differences for *p* < 0.05.

*T*-test analysis revealed significant differences between young-old and old groups for MMSE (*p* < 0.05), Digit Span Forward (*p* < 0.05), CORSI (*p* < 0.01), and N-Back task (*p* < 0.01) (Figure 3). Further analyses were performed within and between groups for sex and education, defined as high (>9 years) and low (≤9 years) level, to see whether cognitive performances of both groups were affected by demographic factors. *T*-tests of inter-groups differences for high/low level of education and sex showed significant differences for education in Digit Span Forward (*p* < 0.05) and Backward (*p* < 0.01), reporting higher accuracy for more educated individuals, and for sex in Digit Span Backward (*p* < 0.05) and COWAT (*p* < 0.05), showing higher accuracy for females in both tasks. For the young-old adults, *t*-test analysis revealed differences between females (*N* = 8) and males (*N* = 10) for Digit Span Backward (*p* < 0.01), showing higher performance accuracy for females, and for CORSI (*p* < 0.05), showing in contrast higher performance accuracy for males. Also, *t*-test analysis indicated significant differences between high (*N* = 9)/low (*N* = 11) level of education for Digit Span Forward (*p* < 0.05) and for COWAT (*p* < 0.01), showing higher performance accuracy for more educated individuals. For the old adults, *t*-test analysis did not show any significant difference for sex and educational level.

Additional analyses were performed for the N-Back task to assess differences in behavioral performance between young-old and old subjects. As we previously found that behavioral performance by using N-Back task was higher for young-old adults compared to old adults, we divided the responses to the stimuli into four categories: hit (target and button press), false alarm (non-target and button press), correct rejection (non-target and no button press), and miss (target and no button press). We performed a two-way ANOVA with factors age-group and N-Back level, and found a significant effect of interaction between the two factors (*p* < 0.05) in accuracy for the 1-Back task only. No significant results were found for 2 and 3-Back task when comparing the two age groups.

### Electrophysiological Results

#### ERPs Responses

**FIGURE 4:**
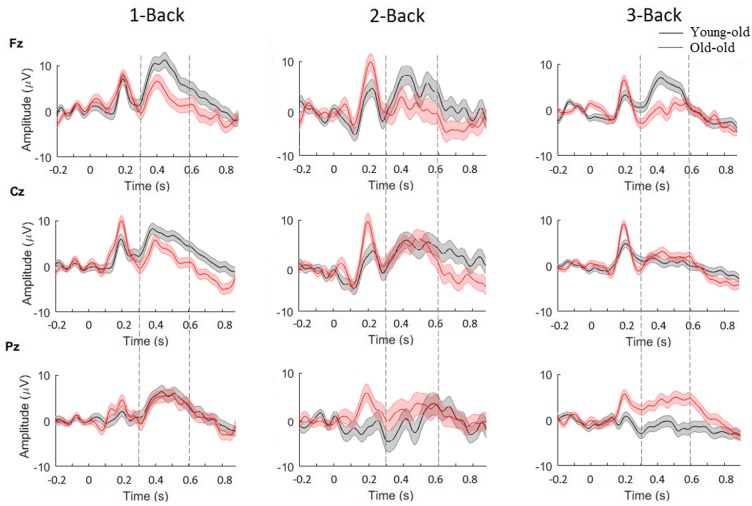
Grand average of P300-ERP component for young-old (black) and old adults (red), for channels Fz, Cz, and Pz. Significance was measured using three-way ANOVA (*p* < 0.05). Error bars indicate SEM.

We analyzed P300-ERPs component by using electrodes located over three main scalp areas: frontal (Fz), central (Cz), and parietal (Pz). Grand-averaged epochs (time window between 250 and 500 ms) for target trials, for each difficulty level of the N-Back task (1, 2, and 3) are shown in Figure 4. A two-way ANOVA (age-group × target) was used to detect significant modulations of P300 magnitude for all three channels (Fz, Cz, Pz). Based on our results, we observed that P300 amplitude changed significantly between young-old and old adults in channel Fz [*F*(1) = 5.5881, *p* < 0.05] and in channels Pz [*F*(1) = 14.0118, *p* < 0.001] for the 3-Back task. Additionally, a comparison between age-groups revealed significant differences in P300 latency in channel Fz for 1-Back [*F*(1) = 6.2955, *p* < 0.05], 2-Back [*F*(1) = 26.0533, *p* < 0.000001] and 3-Back [*F*(1) = 16.1679, *p* < 0.0001], in channel Cz for 1-Back [*F*(1) = 16.8572, *p* < 0.0001], 2-Back [*F*(1) = 16.3562, *p* < 0.0001], and 3-Back [*F*(1) = 10.8322, *p* = 0.001], and in channel Pz for 1-Back [*F*(1) = 16.5398, *p* < 0.0001], 2-Back [*F*(1) = 3.8854, *p* < 0.05], and 3-Back [*F*(1) = 8.5217, *p* < 0.01]. In general, we observed higher P300 amplitudes and smaller latencies for young-old adults compared to old individuals.

Furthermore, we wanted to see whether differences for sex and educational level were present in our sample. By using a two-way ANOVA, with factors sex and target, to detect significant modulations of P300 magnitude for all three channels (Fz, Cz, Pz), we found significant differences between males and females in P300 amplitude for 1-Back in channel Cz [*F*(1) = 7.52, *p* < 0.01] and channel Pz [*F*(1) = 7.43, *p* < 0.01], and in P300 latency for 3-Back in channel Fz [*F*(1) = 9.77, *p* < 0.01] and in channel Cz [*F*(1) = 12.01, *p* < 0.001], indicating higher P300 amplitudes and smaller latencies for females compared to males. Also, performing a two-way ANOVA, with factors educational level and target, we found significant differences between participants with high/low educational level in P300 amplitude for 3-Back in channel Cz [*F*(1) = 5.49, *p* < 0.05] and in channel Pz [*F*(1) = 22.66, *p* < 0.001], and in P300 latency for 1-Back in channel Fz [*F*(1) = 5.36, *p* < 0.05] and channel Cz [*F*(1) = 4.75, *p* < 0.05], and for 2-Back in channel Fz [*F*(1) = 4.39, *p* < 0.05] and channel Cz [*F*(1) = 5.36, *p* < 0.05]. These findings showed higher P300 amplitude and smaller latencies for subjects with a higher educational level. In contrast, by using two three-way ANOVA (sex × age-groups × target, educational level × age-groups × target) that included the interaction of both sex and educational level with age, we did not find any significant difference.

### MRI Results

#### Correlations Between Gray Matter Volume (VBM) and P300 Amplitude and Latency

**FIGURE 5:**
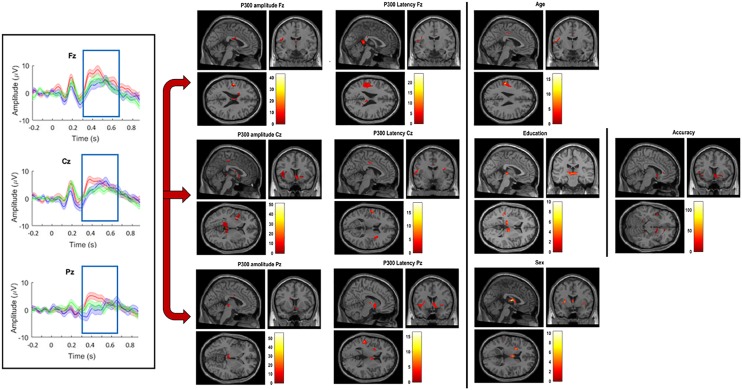
Grand-average of P300 amplitude and latency for channels Fz, Cz, and Pz (left); significant correlations between VBM gray matter and amplitude and latency for channels Fz, Cz, and Pz (middle); significant correlations between VBM gray matter volume and age, education, sex, and accuracy (right). (All significant correlations are shown for *p* < 0.05).

The N-Back P300 amplitudes and latencies of the 16 participants included in the old adults group that underwent an MRI scan were correlated with their gray matter volumes using linear regression analysis, controlling for age, education, sex and TIV. Significant statistical correlations for frontal, central and parietal electrodes separately, and demographics are shown in Table 2 and Figure 5. Our results show significant correlations (uncorrected for FWE, *p* < 0.001) between P300 amplitude in the frontal scalp area (channel Fz) and gray matter volume peak- and cluster levels in the left post-central gyrus (MNI coordinates of the most significant peak: −49.4 −10.3 23; cluster level *k* = 49; peak level *T* = 43.33), in the central area (channel Cz) and gray matter volume peak- and cluster levels in the left lingual gyrus (MNI coordinates of the most significant peak: −15.2 −41.6 −1; cluster level *k* = 63; peak level *T* = 42.06), and similarly (uncorrected for FWE, *p* < 0.01) between the parietal area (channel Pz) and the left thalamus (MNI coordinates of the most significant peak: −14.5 −22.3 2; cluster level *k* = 242; peak level *T* = 28.07) and the thalamus (MNI coordinates of the most significant peak: 1.7 −16.9 2; cluster level *k* = 242; peak level *T* = 22.12). Additionally, we found significant correlations (uncorrected for FWE, *p* < 0.001) between P300 latency in the frontal scalp area (channel Fz) and peak- and cluster levels in the left supramarginal gyrus (MNI coordinates of the most significant peak: −50.7 −27.5 28; cluster level *k* = 106; peak level *T* = 16.92), the central area (channel Cz) and the left post-central gyrus (MNI coordinates of the most significant peak: −54.5 −9.3 19; cluster level *k* = 49; peak level *T* = 18.61), and the left supramarginal gyrus (MNI coordinates of the most significant peak: −51.8 −26.4 28; cluster level *k* = 33; peak level *T* = 14.06), and the parietal area (channel Pz) and the right thalamus (MNI coordinates of the most significant peak: 13.4 −10.5 5; cluster level *k* = 36; peak level *T* = 16.55) and between the parietal area and the temporal thalamus (MNI coordinates of the most significant peak: 8 −4.1 6; cluster level *k* = 36; peak level *T* = 10.71). Moreover, we found significant correlations between N-Back accuracy and peak- and cluster levels (uncorrected for FWE, *p* < 0.001) in the middle temporal gyrus (MNI coordinates of the most significant peak: −53.7 −32.8 7; cluster level *k* = 33; peak level *T* = 120.57). Last, we found significant correlations between age and peak- and cluster levels (uncorrected for FWE, *p* < 0.01) in the left caudate nucleus (MNI coordinates of the most significant peak: − 16.2 −16.4 23; cluster level *k* = 90; peak level *T* = 37.83) and in the thalamus temporal (MNI coordinates of the most significant peak: 2.2 −6.3 13; cluster level *k* = 133; peak level *T* = 10.61), between education and peak- and cluster levels (uncorrected for FWE, *p* < 0.001) in the thalamus (MNI coordinates of the most significant peak: 1.7 −16.9 2; cluster level *k* = 80; peak level *T* = 51.86), and between sex and peak- and cluster levels (uncorrected for FWE, *p* < 0.001) in the left post-central gyrus (MNI coordinates of the most significant peak: −49.4 −10.3 23; cluster level *k* = 39; peak level *T* = 38.55). We did not find significant differences in correlations between P300 amplitudes and latencies and gray matter volume when we considered the three difficulty levels of the N-Back task separately.

**Table 2:** Correlations between gray matter volume, grand-average of N-Back P300 amplitude and latency for channels Fz, Cz, and Pz, accuracy during N-Back task performance, and age, education, and sex in 16 old subjects (uncorrected for FWE, ^∗^*p* < 0.01, ^∗∗^*p* < 0.001).

Variables	MNI coordinates (mm)			*T*–value (peak level)	*K*–value (cluster level)	Labels	%	P uncorrected cluster level
						
	*X*	*Y*	*Z*					
N-Back P300 amplitude Fz	−49.4	−10.3	23	43.33	49	L post-central gyrus	Area 3a, 21%	<0.001^∗∗^
N-Back P300 amplitude Cz	−15.2	−41.6	−1	42.06	63	L lingual gyrus	Subiculum, 6%	<0.001^∗∗^
N-Back P300 amplitude Pz	−14.5	−22.3	2	28.07	242	L thalamus	Motor, 38% Parietal, 34% Premotor, 29%	<0.01^∗^
	1.7	−16.9	2	22.12	242	Thalamus	Prefrontal, 25% Temporal, 29% Visual, 13%	<0.01^∗^
N-Back P300 latency Fz	−50.7	−27.5	28	16.92	106	L supramarginal gyrus	Area PFt (IPL), 38% Area PFop (IPL), 29% Area OP1, 13%	<0.001^∗∗^
N-Back P300 latency Cz	−54.5	−9.3	19	18.61	49	L post-central gyrus	Area 3b, 11% Area 3a, 9%	<0.001^∗∗^
	−51.8	−26.4	28	14.06	33	L supramarginal gyrus	Area PFt (IPL), 50% Area PFop (IPL), 26% Area OP1, 16%	<0.001^∗∗^
N-Back P300 latency Pz	13.4	−10.5	5	16.55	36	R thalamus	Prefrontal, 89% Temporal, 7% Premotor, 5%	<0.001^∗∗^
	8	−4.1	6	10.71	36	Thalamus	Temporal, 54% Prefrontal, 37%	<0.001^∗∗^
N-Back accuracy	−53.7	−32.8	7	120.57	33	Middle tempral gyrus	Area PFcm (IPL), 4%	<0.001^∗∗^
Age	−16.2	−16.4	23	37.83	90	L caudate nucleus	Thalamus, prefrontal, 10% Visual, 8% Premotor, 7%	<0.01^∗^
	2.2	−6.3	13	10.61	133	Thalamus	Temporal, 12%	<0.01^∗^
Education	1.7	−16.9	2	35.16	80	Thalamus	Prefrontal, 25% Temporal, 29% Visual, 13%	<0.001^∗∗^
Sex	−49.4	−10.3	23	38.55	39	L post-central gyrus	Area 3a, 21%	<0.001^∗∗^

## Discussion

The main purpose of our study was to explore the effect of aging, sex and education on cognitive test performance and visual P300-ERP component recorded during N-Back task, and the novelty was to correlate the P300 characteristics with gray matter volume using the VBM technique for whole brain. We first assessed the cognitive test performance and P300 amplitude and latency for both age sub-groups, and second we examined the relationship between P300 characteristics and gray matter volume, and the correlations between age, education, sex, and gray matter volume, for 16 healthy normal aging individuals that underwent an MRI scan. We expected significant differences in cognitive and neurophysiological results between age-groups, sex and education, significant correlations between P300 amplitude and latency and gray matter volume in frontal and parietal lobes. In addition, motivated by Schippling et al. (2017) who investigated atrophy level in normal aging and correlated brain volume with age, sex, education, and TIV, we also expected to find significant correlations between our sample’s demographics and gray matter volume.

To demonstrate the first hypothesis, we performed a *t*-test comparing young-old and old groups on their cognitive tests performance and demographic information. We administered a battery of cognitive tests that included MMSE, Digit Span Forward and Backward, COWAT, VAT, Stroop, TMT-A and B, N-Back, TOVA, CORSI, and RAVEN tests. Encouraged by the study of Duñabeitia et al. (2018) and Wiederholt et al. (1993), which revealed that educational level and sex can affect cognitive tests performance, we investigated both parameters to see whether they could explain our behavioral results. We performed two-way ANOVA tests crossing N-Back task performance and age-groups, sex and educational level. At cognitive level, significant differences were found between the two age-groups in MMSE, CORSI, and N-Back tasks. These results are consistent with Christensen (2001) who showed changes over adult lifespan in cognitive tests performance. Furthermore, we showed that sex and education had a significant effect on cognitive performance for both young-old and old subjects in Digit Span Backward and COWAT for sex, reporting higher performance accuracy for females in both tests, and for Digit Span Forward and Backward for education, revealing higher accuracy for more educated individuals. Moreover, our results indicated that sex and education also affected cognitive performance of young-old adults separately, reporting higher performance accuracy for females in Digit Span Backward (short-term memory) task, and for males in CORSI (spatial memory) task, and higher performance accuracy for more educated individuals in Digit Span Forward (short-term memory) and COWAT (verbal fluency) tasks compared to lower educated individuals. These findings add weight to the hypothesized effect of education on cognitive reserve to curb the negative effect of aging on one’s cognitive abilities (Stern, 2009).

Additionally, we examined P300 amplitude and latency, recorded during N-Back task performance for both healthy elderly groups. Although some studies promoted the P300 component as a biomarker of (the degree of) impairment in healthy elderly at risk of developing AD (Newsome et al., 2013), we did not follow the same direction as the topic is still controversial. In contrast, studies that investigated P300 amplitude and latency in normal aging are abundant (Polich and Kok, 1995; Polich, 2007; Redmond et al., 2012; van Dinteren et al., 2014; Lubitz et al., 2017) albeit that for P300 latency a stronger relation to age was observed than for P300 amplitude (Derambure et al., 1993; Barrett et al., 1999; Reuter et al., 2013). Although latency seemed to more promising in revealing aging effects, we explored both P300 features – amplitude and latency – since the P300 component is the one that is most strongly modulated by the N-Back task we administered (Watter et al., 2001), and where the P300 amplitude is modulated by attention and memory load, while latency by performance of matching (Chen et al., 2008).

Our results revealed differences in P300 amplitude between age-groups, showing a higher P300 amplitude and smaller P300 latency for young-old compared to old adults in all three N-Back levels, providing indications about decline in memory processes. Both analyses were consistent with the results of Polich (2007) and Lubitz et al. (2017). Furthermore, we replicated the same analysis for cognitive testing, including sex and level of education, for the P300-ERP component. Higher P300 amplitudes and smaller P300 latencies were found for females compared to males. Also, higher P300 amplitudes and smaller P300 latencies were observed for individuals that were more educated compared to less educated ones. We hypothesized that females process visual information differently from males. These results support the data shown by Steffensen et al. (2008) where females were characterized by a greater P300 response compared to males during an object recognition task, and are in line with those of Angel et al.’s (2010) where the effect of age on ERPs responses was shown to be smaller for higher educated individuals compared to lower educated ones during a word-stem cued-recall task. Also in this case, as for the cognitive tests results, education level seemed to compensate for the effect of aging, and therefore to play a protective role as claimed by the cognitive reserve hypothesis. Higher educational levels were shown to be associated with greater synaptic density and more complex networks (Kramer et al., 2004) and a higher cognitive reserve, e.g., due to a higher educational background, could compensate for neurocognitive deficits, by the ability to recruit alternative brain networks, and in this way maintain a high cognitive performance (Park and Reuter-Lorenz, 2009).

To validate our second hypothesis, we used the 16 old participants that underwent an MRI scan, and correlated their N-Back P300 amplitude and latency with the gray matter volume. To the best of our knowledge, there are no studies published on neuroanatomical correlates of P300 in healthy older adults during a WM task, in our specific case an N-Back task. Only one study examined the correlation between P300 amplitude and gray matter volume of specific ROIs during attentional tasks (Ford et al., 1994). The results we reported showed significant correlations between gray matter volume, analyzed for the whole brain, instead of ROIs, using VBM, and P300 amplitude and latency for frontal, central and parietal electrodes. In particular, we found significant correlations between frontal, central and parietal P300 amplitude and -latency and gray matter volume in left parietal areas (left post-central gyrus and left supramarginal gyrus), temporal (middle temporal gyrus), and occipital (left lingual gyrus) lobes and the thalamus. Also, we found a larger gray matter volume related to higher P300 amplitude and shorter latency. These data are consistent with studies that showed age-related gray matter volume loss in different substructures, such as medial, parietal and temporal structures, in older adults (Grieve et al., 2005; Hutton et al., 2009; Giorgio et al., 2010; Terribilli et al., 2011; Schippling et al., 2017), and in line with the study of Ford et al. (1994) that showed significant correlations between P300 and gray matter volume in parietal lobe when P300 was recorded during auditory attention tasks. The brain regions we found to be significantly correlated with the P300 component and demographics of our elderly individuals also have an important role in regulating cognitive and neurophysiological functions. In particular, the thalamus is known to be involved in the process and integration of neocortical inputs and outputs (Fama and Sullivan, 2015) and its connectivity shown to decrease with age and to be most strongly reduced in MCI and AD patients causing besides personality and mood disorders, also arousal and sleep complaints. Furthermore, the left post-central gyrus, which is the core of the somatosensory network (Tomasi and Volkow, 2011), has been shown to become thinner with age (Salat et al., 2004). The middle temporal gyrus has been associated with the recognition of faces and access to word meaning (Acheson and Hagoort, 2013), although its exact function is still largely unknown, and shown to be affected by age-related volume loss (Raz et al., 2004). Also, the left lingual gyrus, linked to processing vision and encoding visual memories, has been found to be modulated by age (Swierkot and Rajah, 2018). Similarly, the supramarginal gyrus (Sussman et al., 2016), related with phonological word choices and language perception and processing, and the caudate nucleus (Jiji et al., 2013), related to several executive functions such as memory, learning, inhibitory control, etc., have both been reported to decrease with age. Last, our results showed that also demographics such as age, education and sex, correlate with gray matter volume, especially in the parietal lobe and thalamus, revealing a smaller gray matter volume with increasing age (negative correlation), but a larger gray matter volume for more educated older individuals and females (positive correlations). The latter supports the results of several studies (Raz et al., 1997; Good et al., 2001; Steffener et al., 2014; Schippling et al., 2017) that provided evidence of negative correlations between age, and positive relationships between sex and education, and gray matter volume.

## Conclusion

Our study demonstrated that age, sex and educational level affect cognitive, neurophysiological (EEG) and structural (MRI) responses in healthy older adults, supporting previous findings. Among older adults, only those with a higher level of education revealed a better cognitive performance and a larger P300 amplitude and shorter P300 latency. However, as to sex the affect was more complicated: a larger P300 amplitude and shorter P300 latency for females compared to males, and a higher short-term memory task accuracy for females, but conversely a better spatial memory task performance for males. Additionally, exploring the relationship between anatomical and temporal characteristics, we observed significant correlations between the P300 component, age, sex and education, and gray matter volume in normal aging individuals that performed an N-Back task. These preliminary findings call for further investigation also by using traditional biomarkers of neurodegeneration such as amyloid PET, to control for participants that are already in a preclinical phase, and by implicating additional cognitive reserve factors such as bilingualism, professional attainment and leisure activities.

## Ethics Statement

This study was carried out in accordance with the recommendations of “EC Research, Ethical Committee UZ/KU Leuven” with written informed consent from all subjects. All subjects gave written informed consent in accordance with the Declaration of Helsinki. The protocol was approved by the “Ethical Committee UZ/KU Leuven” of Gasthuisberg Hospital.

## Author Contributions

VP and MVH designed the study. VP acquired the data, processed the neuroimaging data, and performed the statistical analysis. JT and BS helped with the participants’ recruitment. All authors contributed to data interpretation and to the writing of the manuscript.

## Conflict of Interest Statement

The authors declare that the research was conducted in the absence of any commercial or financial relationships that could be construed as a potential conflict of interest.

## Footnotes

1 www.fil.ion.ucl.ac.uk/spm

2 nl.mathworks.com/products/matlab/

3 http://www.gin.cnrs.fr/AAL
